# The MALAT1-EZH2 axis regulates PRC2 activity and promotes the mesenchymal phenotype in pediatric atypical teratoid/rhabdoid tumors

**DOI:** 10.1007/s11060-026-05539-x

**Published:** 2026-03-31

**Authors:** Melisa Gurbuz, Cagla Tekin, Melis Ercelik, Sevin Avsar Koc, Feray Kockar, Pınar Eser, Mevlut Ozgur Taskapilioglu, Gulcin Tezcan, Burcu Erbaykent, Ahmet Bekar, Hasan Kocaeli, Mine Ozsen, Pınar Karabaglı, Hakan Karabaglı, Buşra Yaprak Bayrak, Volkan Etus, Berrin Tunca

**Affiliations:** 1 Department of Medical Biology, Faculty of Medicine, Bursa Uludag University, Gorukle, Bursa, 16059 Türkiye; 2 Department of Molecular Biology and Genetics, Arts and Science Faculty, Balikesir University, Balikesir, Türkiye; 3 Department of Neurosurgery, Faculty of Medicine, Bursa Uludag University, Bursa, Türkiye; 4 Department of Neurosurgery, Faculty of Medicine, Istanbul Yeni Yuzyıl University, Istanbul, Turkey; 5 Department of Fundamental Sciences, Faculty of Dentistry, Bursa Uludag University, Bursa, Türkiye; 6 Division of Molecular Biology & Genetics, School of Arts & Sciences, Bursa Uludag University, Bursa, Türkiye; 7 Department of Pathology, Faculty of Medicine, Bursa Uludag University, Bursa, Türkiye; 8 Department of Pathology, Faculty of Medicine, Muğla Sıtkı Koçman University, Muğla, Türkiye; 9 Department of Neurosurgery, Faculty of Medicine, Muğla Sıtkı Koçman University, Muğla, Türkiye; 10 Department of Pathology, Faculty of Medicine, Kocaeli University, Kocaeli, Türkiye; 11 Department of Neurosurgery, Kocaeli University Faculty of Medicine, Kocaeli, Türkiye

**Keywords:** Atypical teratoid/rhabdoid tumor (AT/RT), EZH2, MALAT1, lncRNA, Epigenetics

## Abstract

**Background:**

Atypical teratoid/rhabdoid tumors (AT/RT) are aggressive pediatric CNS malignancies characterized by SMARCB1 loss, which leads to the dysregulated expression of Enhancer of Zeste Homolog 2 (EZH2), a key catalytic component of the Polycomb Repressive Complex 2 (PRC2). This dysregulation results in aberrant trimethylation of histone H3 at lysine 27 (H3K27me3), driving tumor progression. While EZH2 inhibitors like tazemetostat are in clinical use, their efficacy remains limited, necessitating a deeper understanding of PRC2 regulation. We investigated the role of long non-coding RNAs (lncRNAs) in modulating the EZH2-PRC2 axis in AT/RT.

**Methods:**

Expression levels of lncRNAs (MALAT1, ANRIL, KCNQ1OT1) were analyzed via RT-PCR in 10 archival AT/RT patient tissues. RNA immunoprecipitation (RIP) was performed to identify direct interactions with EZH2. The functional impact of MALAT1 inhibition on H3K27me3 levels, mesenchymal markers (CDH2, TWIST, ZEB1), and tumorigenic behaviors (migration, invasion, sphere formation) was evaluated in vitro.

**Results:**

MALAT1, ANRIL, and KCNQ1OT1 were significantly overexpressed in AT/RT tissues (*p* < 0.05). RIP assays revealed that only MALAT1 directly interacts with EZH2 in DAOY and primary AT/RT cells. MALAT1 knockdown significantly reduced H3K27me3 levels (*p* < 0.05) and markedly impaired cell migration, invasion, and sphere-forming capacity. These phenotypic changes were associated with the downregulation of key mesenchymal markers (CDH2, TWIST, ZEB1).

**Conclusions:**

Our findings identify MALAT1 as a critical epigenetic regulator in AT/RT that interacts with EZH2 to maintain the PRC2-mediated repressive landscape. Targeting the MALAT1-EZH2 axis provides a novel translational perspective to enhance the efficacy of epigenetic therapies in AT/RT.

**Graphical abstract:**

**Figure:**
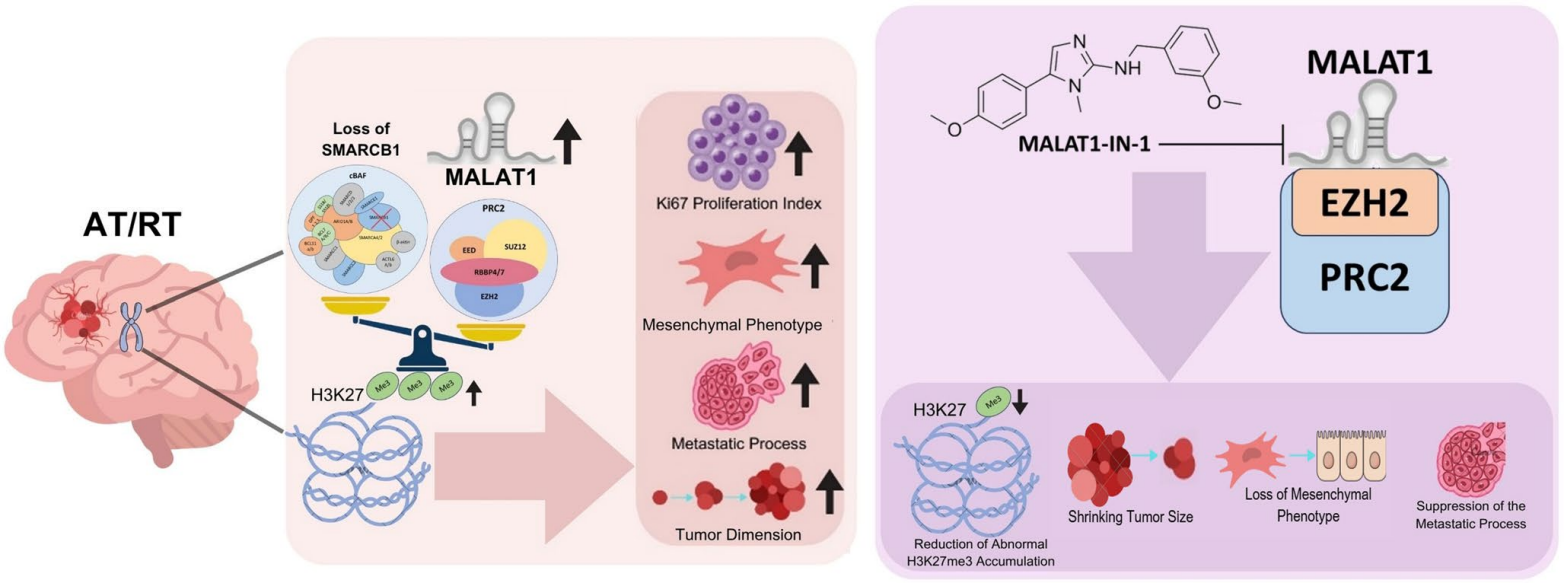
Proposed Mechanism of MALAT1-Mediated Epigenetic Regulation in AT/RT. This study elucidates the oncogenic role of the long noncoding RNA MALAT1 in Atypical Teratoid/Rhabdoid Tumors (AT/RT). Pathogenesis: In the context of SMARCB1 loss, MALAT1 is significantly upregulated, leading to enhanced recruitment of the EZH2/PRC2 complex. This interaction results in an aberrant increase in H3K27me3 levels, driving tumor progression by increasing the Ki67 proliferation index, promoting a mesenchymal phenotype, and accelerating metastatic processes and tumor growth. Therapeutic Intervention: Inhibition of MALAT1 (via MALAT1-IN-1) disrupts its interaction with the PRC2 complex. This targeted intervention leads to a reduction in abnormal H3K27me3 accumulation, subsequently shrinking tumor size, reversing the mesenchymal phenotype, and suppressing metastatic activity. Overall, the findings highlight MALAT1 as a viable epigenetic target to counteract the aggressive progression of AT/RT.

**Supplementary Information:**

The online version contains supplementary material available at 10.1007/s11060-026-05539-x.

## Key points

MALAT1 is significantly upregulated in AT/RT and correlates with aggressive clinicopathological features.

MALAT1 physically interacts with EZH2, sustaining the H3K27me3 repressive landscape in patient-derived cells.

Inhibition of the MALAT1-EZH2 axis reverses the mesenchymal phenotype and impairs tumor sphere growth.

EZH2 inhibition triggers a compensatory increase in MALAT1, suggesting a novel feedback loop and resistance mechanism.

Dual targeting of MALAT1 and EZH2 represents a promising therapeutic strategy for pediatric rhabdoid tumors.

**Supplementary Information:**

The online version contains supplementary material available at 10.1007/s11060-026-05539-x.

## Introduction

Atypical teratoid/rhabdoid tumors (AT/RTs) are highly aggressive pediatric CNS malignancies, primarily diagnosed in children under age three. Despite intensive multimodal therapies, the prognosis remains poor due to surgical constraints and the long-term neurocognitive toxicity of radiation in infants [1]. The hallmark of AT/RT is the biallelic loss of SMARCB1 (INI-1) on chromosome 22, a core subunit of the SWI/SNF chromatin-remodeling complex [2]. While AT/RTs are molecularly classified into TYR, SHH, and MYC subgroups, SMARCB1 loss remains the unifying driver, with SMARCA4 mutations occurring in rare cases [3].

Physiologically, the SWI/SNF complex antagonizes Polycomb Repressive Complex 2 (PRC2). SMARCB1 deficiency disrupts this homeostatic balance, leading to unchecked activity of EZH2, the catalytic subunit of PRC2. This results in aberrant H3K27me3 accumulation at tumor suppressor promoters, thereby silencing differentiation-related genes and driving oncogenesis [4]. In other pediatric embryonal CNS tumors, such as the SHH subtype of medulloblastoma (represented by the DAOY cell line), a similar epigenetic dysregulation via EZH2 is observed, highlighting shared pathogenic mechanisms [5–7]. Although driven by distinct genetic alterations (SMARCB1 loss vs. SHH activation), AT/RT and SHH-medulloblastoma converge on EZH2-mediated repressive landscapes and shared developmental arrest programs [7, 8]. Thus, the EZH2/PRC2 axis represents a common epigenetic vulnerability in these diverse pediatric CNS tumors [8].

The EZH2 inhibitor tazemetostat has shown antineoplastic activity in SMARCB1-deficient tumors, yielding objective responses in pediatric trials [9]. However, clinical success remains limited, necessitating the investigation of alternative regulatory mechanisms, such as lncRNA-PRC2 interactions, to improve therapeutic targeting. lncRNAs, including MALAT1, ANRIL, and KCNQ1OT1, are consistently dysregulated in CNS malignancies and are known to modulate gene silencing through direct EZH2 binding [10–15]. Specifically, these lncRNAs act as molecular scaffolds that recruit PRC2 to specific genomic loci, driving tumor progression and therapy resistance in malignant brain tumors [14, 15].

In this study, we investigated the clinical significance of these candidate lncRNAs in AT/RT patient tissues and their physical association with EZH2. Furthermore, using primary AT/RT and DAOY cell models—the latter serving as a representative model for EZH2-mediated epigenetic dysregulation—we evaluated whether targeting these lncRNAs could suppress tumor aggressiveness and mesenchymal features, comparing their efficacy with tazemetostat.

## Materials and methods

### Patient samples

This study was approved by the Bursa Uludag University Faculty of Medicine Ethics Committee (Approvals: 2020-12/16, 2023-26/19). Ten histologically confirmed primary AT/RT tissue samples, resected prior to chemo-radiotherapy between 2015 and 2021, were retrospectively collected from three centers (Bursa Uludag, Selcuk, and Kocaeli Universities). Exclusion criteria included biopsy-only cases, family history of glioma, concurrent malignancies, or perioperative mortality (< 30 days). Non-neoplastic cortical tissues from three epilepsy patients undergoing temporal lobectomy served as controls. The Ki-67 proliferation index was quantified by assessing at least 1000 tumor cells in hotspot areas at ×400 magnification.

### RNA extraction and RT–qPCR

RNA was extracted from 5 to 10 μm FFPE sections using a commercial kit (Nucleogene, Türkiye) following pathological confirmation of tumor regions. RNA purity (A260/280 ratio 2.0–2.2) was verified via spectrophotometry (Beckman Coulter DU-730). Total RNA (500 ng) was reverse-transcribed using a high-capacity cDNA synthesis kit (Thermo Fisher Scientific). Expression of MALAT1, ANRIL, and KCNQ1OT1 was quantified via RT–qPCR (Thermo Fisher Scientific, StepOne Real-Time PCR System) using the 2^Delta\Delta Ct method, with GraphPad Prism 8.4.2 (RRID: SCR_002798) as the internal control.

### Cell culture

**Fig. 1:**
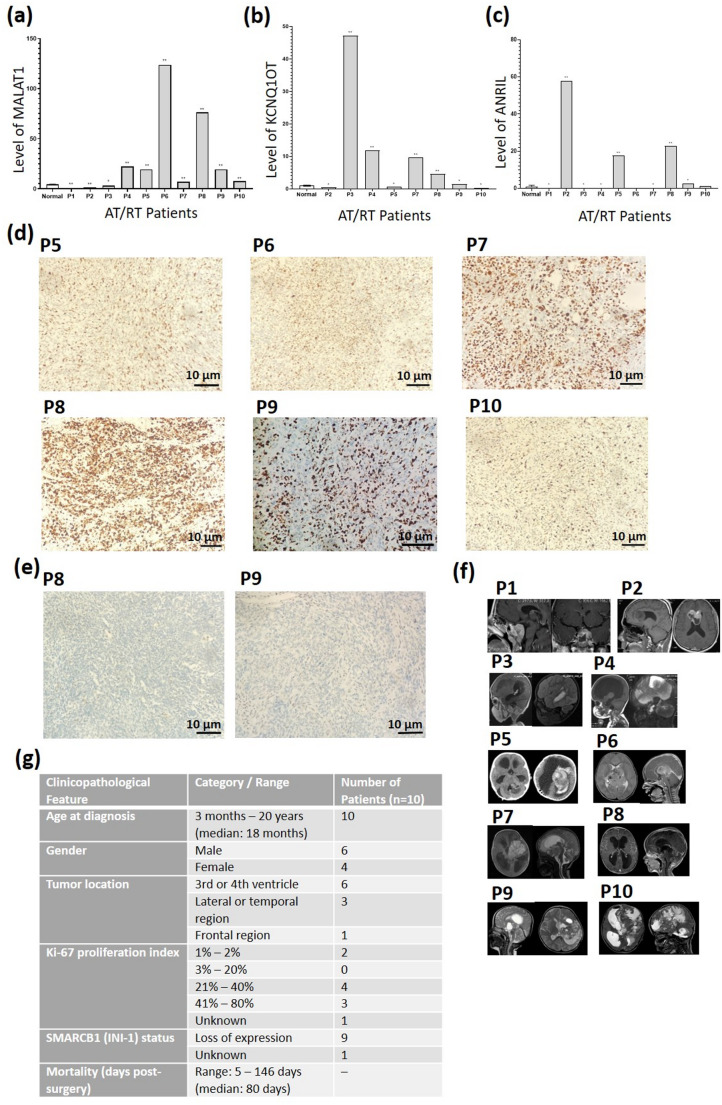
Upregulation of MALAT1, ANRIL, and KCNQ1OT1 in AT/RT tissues. (**a–c**) RT–qPCR analysis of MALAT1, KCNQ1OT1, and ANRIL expression in AT/RT tumors relative to non-tumor epilepsy controls, normalized to GAPDH (*p < 0.05, **p < 0.0001). (**d**) Representative Ki-67 IHC staining in AT/RT tissues (P5–P10). (**e**) IHC staining showing the loss of nuclear INI1 (SMARCB1) expression in a representative patient (P8) from paraffin blocks of patients in whom lncRNA expression was evaluated, and in tissue from an AT/RT patient (P9), where cell culture analyses were performed. (**f**) MRI images showing tumor localization in AT/RT patients. Control samples were normalized to 1 by ΔΔCt; error bars are omitted for controls. (**g**) Summary of clinicopathological characteristics of AT/RT patients (To preserve patient confidentiality, clinicopathological data are presented as grouped or summary statistics rather than as individual-level information.) Individual patient data correlating MALAT1 levels with Ki-67 indices and anatomical tumor locations are detailed in Table S1

The primary AT/RT cell line was established from a pediatric patient (a 9-month-old male) at our institution. The diagnosis and authenticity of the primary cells were confirmed by a specialized pathologist through immunohistochemical staining (IHC), demonstrating a total loss of nuclear INI1 (SMARCB1) expression (Fig. 1). Cells were maintained in DMEM/F-12 supplemented with L-glutamine, 10% FBS, and sodium pyruvate. The DAOY medulloblastoma line (SHH subtype) (RRID: CVCL_1167) was cultured in DMEM with similar additives. DAOY served as a representative model for EZH2-mediated epigenetic dysregulation in pediatric CNS tumors [5, 6], while primary cells were used to validate tumor-specific heterogeneity.

### RNA immunoprecipitation (RIP)

RIP was performed to assess EZH2–lncRNA interactions using an RNA-binding protein immunoprecipitation kit (Millipore #17–701) and anti-EZH2 antibody (Thermo Fisher Scientific Cat# PA5-17569, RRID: AB_10986489) (1:50). Immunoprecipitated RNA was analyzed for MALAT1, ANRIL, and KCNQ1OT1 expression via RT–qPCR (TaqMan assays, Invitrogen).

### Proliferation and immunofluorescence

Cell viability was measured via MTT assay (Thermo Fisher Scientific) following 7-day treatment with tazemetostat or MALAT1-IN-1. Sub-cytotoxic doses (< IC30) were selected for subsequent assays. For H3K27me3 evaluation, cells were treated with 2.5 μm tazemetostat (7 days) or 5 μm MALAT1-IN-1 (24 h), stained with Alexa Fluor 488-conjugated anti-H3K27me3 (1:800) (Cell Signaling Technology Cat# 98316, RRID: AB_2943245), and counterstained with DAPI. Nuclear localization was quantified using an EVOS M5000 system. Treatment durations were optimized based on the distinct kinetics of each inhibitor: a 7-day period was employed for the epigenetic modulator tazemetostat to allow for cumulative changes in histone methylation and cell cycle progression [16, 17], while a 24-hour period was utilized for MALAT1-IN-1 to capture its acute impact on lncRNA-mediated processes, consistent with established inhibitory profiles for these classes of compounds [18, 19].

### Migration and invasion assays

Wound healing assays were performed on confluent monolayers; wound closure was monitored using ImageJ (RRID: SCR_003070) after treatment under serum-reduced conditions. Invasion and migration were further evaluated using Matrigel-coated (1:6) or uncoated Transwell chambers (3-µm pore; Millipore). Pretreated cells (5 × 10⁴) were seeded in the upper chamber, with 10% FBS as a chemoattractant. After overnight incubation, invaded/migrated cells were Giemsa-stained and counted across five random fields (40x magnification) using an EVOS M5000 imaging system.

### Sphere formation and viability

DAOY and primary AT/RT cells were seeded in ultra-low-attachment plates to form spheres. Following 7-day inhibitor treatment, sphere size was measured, and viability was assessed via calcein-AM/propidium iodide staining at 488/520 nm and 535/615 nm, respectively, using an EVOS M5000 imaging system.

### Gene expression analysis

Total RNA was isolated for RT–qPCR analysis of P21 (CDKN1A), P27 (CDKN1B), CDH2, TWIST1, ZEB1, and candidate lncRNAs (MALAT1, ANRIL, and KCNQ1OT1). Relative expression was calculated using the ΔΔCt method.

### Statistical analysis

Data (presented as mean ± SE) were analyzed using GraphPad Prism 8.4.2 (RRID: SCR_002798) via one-way/two-way ANOVA or t-tests, as appropriate. Significance was set at *p* < 0.05.

## Results

### The expression of the lncRNAs MALAT1, ANRIL and KCNQ1OT1 was upregulated in AT/RT tissues

The expression of PRC2-associated lncRNAs—MALAT1, ANRIL, and KCNQ1OT1—was analyzed via RT-PCR in AT/RT (*n* = 10) and non-tumor epilepsy (*n* = 3) tissues. MALAT1 was significantly upregulated in 70% of patients, showing a 2- to 120-fold increase compared to controls (Fig. 1a). KCNQ1OT1 and ANRIL were significantly elevated in 50% (2- to 47-fold; Fig. 1b) and 40% (3- to 53-fold; Fig. 1c) of patients, respectively. When correlated with clinical parameters, high expression levels of these lncRNAs were associated with aggressive markers (Fig. 1g). A Ki67 proliferation index > 50% was observed in 3/7 patients with high MALAT1, 2/5 with high KCNQ1OT1, and 1/4 with high ANRIL levels (Fig. 1d). Furthermore, given that medial tumor locations (e.g., 3rd and 4th ventricles) are linked to poorer prognosis compared to lateral regions (Fig. 1f, g) [20], our data revealed a potential prognostic link: most patients with elevated lncRNA levels (6/7 for MALAT1, 5/5 for KCNQ1OT1, and 3/4 for ANRIL) presented with ventricular involvement (Fig. 1a-c, e). These findings suggest that elevated MALAT1, ANRIL, and KCNQ1OT1 expression may parallel aggressive clinico-pathological features and poor prognosis in AT/RT. To investigate the clinical relevance of MALAT1 in AT/RT, we first analyzed its expression levels and the clinicopathological characteristics of the patient cohort. The demographic data, tumor localization, Ki-67 proliferation indices, and relative MALAT1 expression levels for all ten patients (P1–P10) are summarized in Supplementary Table 1.

### MALAT1 binds to Polycomb Repressive Complex 2 (PRC2) via EZH2

**Fig. 2:**
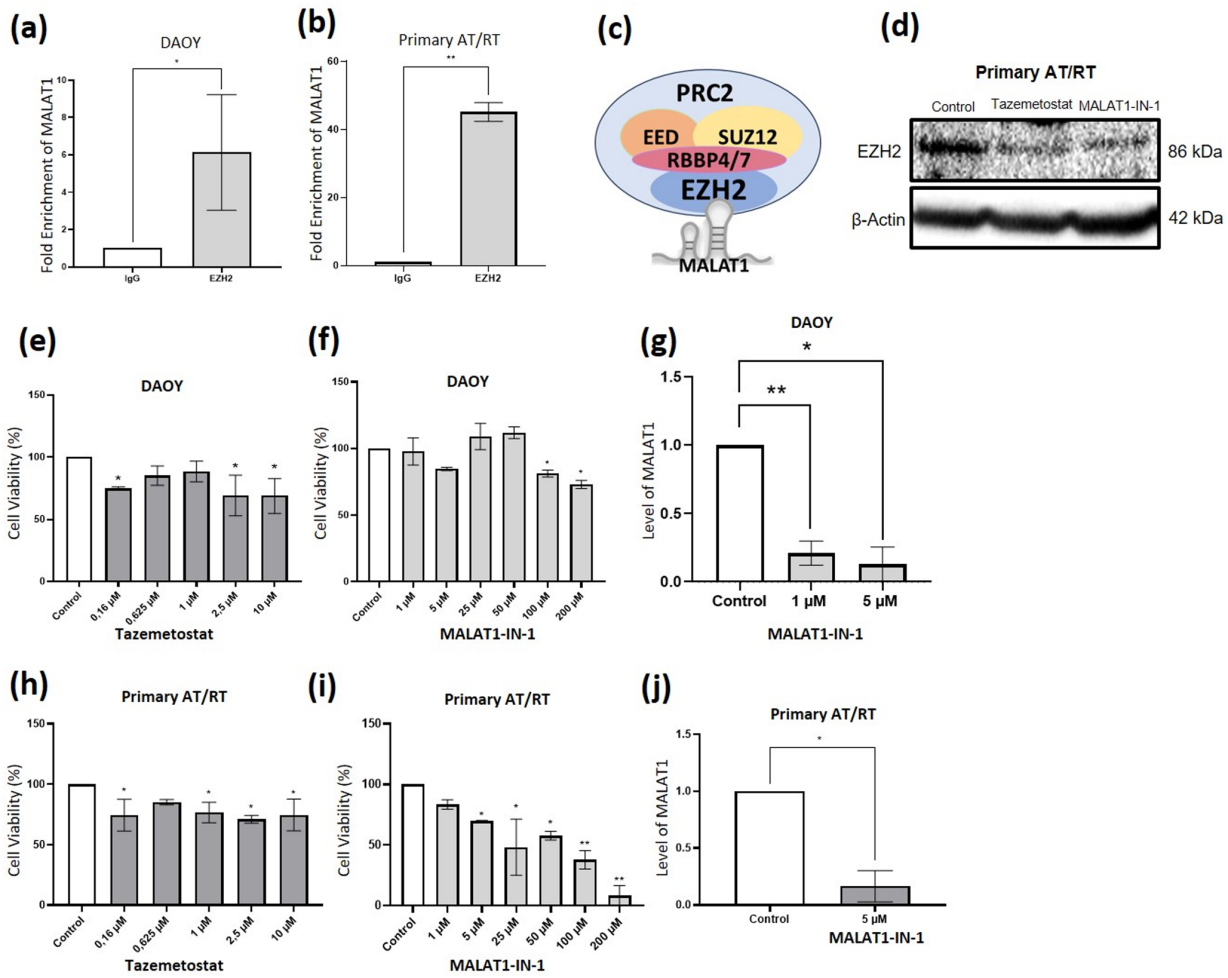
MALAT1 interacts with EZH2 and regulates PRC2 in AT/RT. (**a**,** b**) RIP analysis showing MALAT1 enrichment in DAOY and primary AT/RT cells using EZH2 antibody versus IgG control (*p < 0.05, **p < 0.0001). (**c**) Schematic of MALAT1–PRC2 interaction. (**d**) Western blot of EZH2 protein in primary AT/RT cells treated with tazemetostat or MALAT1-IN-1; β-actin served as loading control (EZH2: 86 kDa; β-actin: 42 kDa). (**e**,** f**) MTT assays showing effects of tazemetostat and MALAT1-IN-1 on DAOY cell proliferation. (**g**) MALAT1 expression in DAOY cells after 1 and 5 µM MALAT1-IN-1 treatment determined by RT–qPCR. (**h**,** i**) MTT assays showing effects of tazemetostat and MALAT1-IN-1 on primary AT/RT cell proliferation (*p < 0.05, **p ≤ 0.0001). (**j**) MALAT1 expression in primary AT/RT cells after 5 µM MALAT1-IN-1 treatment. Controls: untreated or vehicle-treated (DMSO) cells. Control values were normalized to 1 by ΔΔCt; error bars are omitted for controls

PRC2-mediated tumorigenesis is driven by increased EZH2 activity, yet interactions between PRC2 and lncRNAs remain unclear in AT/RT [21, 22]. RIP analysis using EZH2 antibodies demonstrated significant enrichment of MALAT1 in DAOY (6.13-fold; *p* < 0.05) and primary AT/RT cells (45.17-fold; *p* < 0.0001), but not in IgG controls (Fig. 2a, b), indicating direct MALAT1–EZH2 interaction (Fig. 2c). ANRIL and KCNQ1OT1 were not detected in EZH2 immunoprecipitates. Western blot analysis showed that both MALAT1-IN-1 and tazemetostat reduced EZH2 protein levels compared with untreated controls, with comparable degrees of downregulation (Fig. 2d). These findings indicate that MALAT1 directly associates with PRC2 via EZH2 and that MALAT1 inhibition suppresses EZH2 expression.

### Effects of EZH2 and MALAT1 inhibition on cell proliferation

To compare the impact of EZH2 and MALAT1 suppression, DAOY and primary AT/RT cells were treated with tazemetostat or MALAT1-IN-1, and proliferation was assessed by MTT assay. In DAOY cells, 2.5 µM tazemetostat reduced proliferation by ~ 30% at day 7 (*p* < 0.05; Fig. 2e), while 5 µM MALAT1-IN-1 reduced proliferation by ~ 14% at 24 h (Fig. 2f). DAOY cells retained ~ 70% viability even at high MALAT1-IN-1 concentrations (100–200 µM). MALAT1 expression was significantly suppressed by 1 µM (*p* ≤ 0.0001) and more effectively by 5 µM MALAT1-IN-1 (*p* < 0.05) (Fig. 2g). In primary AT/RT cells, 2.5 µM tazemetostat (7 days) and 5 µM MALAT1-IN-1 (24 h) each reduced proliferation by ~ 30% (Fig. 2h, i). Based on IC₃₀ values, these concentrations were selected as optimal for subsequent experiments (Fig. 2j).

### Cell-type-dependent regulation of PRC2 functions by MALAT1

**Fig. 3:**
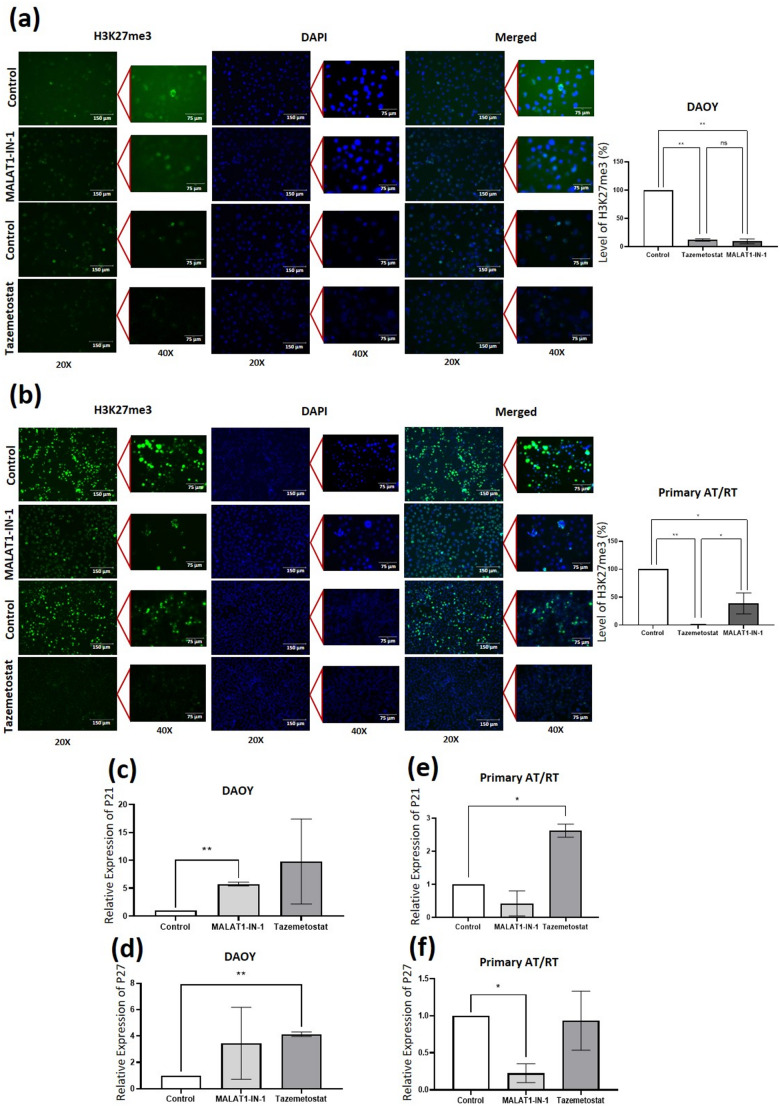
EZH2 and MALAT1 inhibition differentially regulate PRC2-mediated epigenetic repression. DAOY and primary AT/RT cells were treated with tazemetostat (2.5 µM) or MALAT1-IN-1 (5 µM). (**a**,** b**) Immunofluorescence analysis of H3K27me3 (green) in (**a**) DAOY and (**b**) primary AT/RT cells. Scale bars: 150 μm (20×), 75 μm (40×). In DAOY cells, EZH2 and MALAT1 inhibition similarly reduced H3K27me3 (*p* > 0.05), whereas in primary AT/RT cells EZH2 inhibition caused a greater reduction than MALAT1 inhibition (*p* < 0.05). (**c–f**) RT–qPCR analysis of P21 and P27 expression in DAOY (**c**,** d**) and primary AT/RT cells (**e**,** f**), normalized to GAPDH. P21 and P27 increased in DAOY cells after both treatments, but no significant changes were detected in primary AT/RT cells. Controls: vehicle-treated (DMSO) cells. Control values were normalized to 1 by ΔΔCt; error bars omitted. *p < 0.05, **p ≤ 0.0001

The core function of PRC2 is H3K27me3 production, a process regulated by subunit-nucleosome localization and interactions with molecules like MALAT1 [11, 22]. In DAOY cells, EZH2 and MALAT1 inhibition reduced H3K27me3 levels to 11.99% and 9.65%, respectively (*p* < 0.0001). The lack of significant difference between these treatments (*p* > 0.05) suggests that MALAT1 and EZH2 contribute similarly to PRC2-mediated methylation in this context (Fig. 3a). Conversely, in primary AT/RT cells, EZH2 inhibition caused a near-complete loss of H3K27me3 (1.31%), while MALAT1 inhibition resulted in a more moderate reduction (38.97%; *p* < 0.05), indicating a distinct regulatory role for MALAT1 in AT/RT (Fig. 3b). Consistent with the growth inhibition observed, EZH2 inhibition increased p21 and p27 levels in DAOY cells (Fig. 3c, d). In primary AT/RT cells, a similar trend was observed for p21 following EZH2 inhibition, although the induction of these cell cycle inhibitors by MALAT1-IN-1 was less pronounced than in the DAOY model (Fig. 3e). These findings, consistent across replicates, demonstrate that MALAT1-mediated regulation of H3K27me3 and downstream targets like P21/P27 is cell-type-dependent, influencing epigenetic landscapes in specific cellular contexts [23–25].

### MALAT1 inhibition suppressed tumor aggressiveness similarly to EZH2 inhibition

**Fig. 4:**
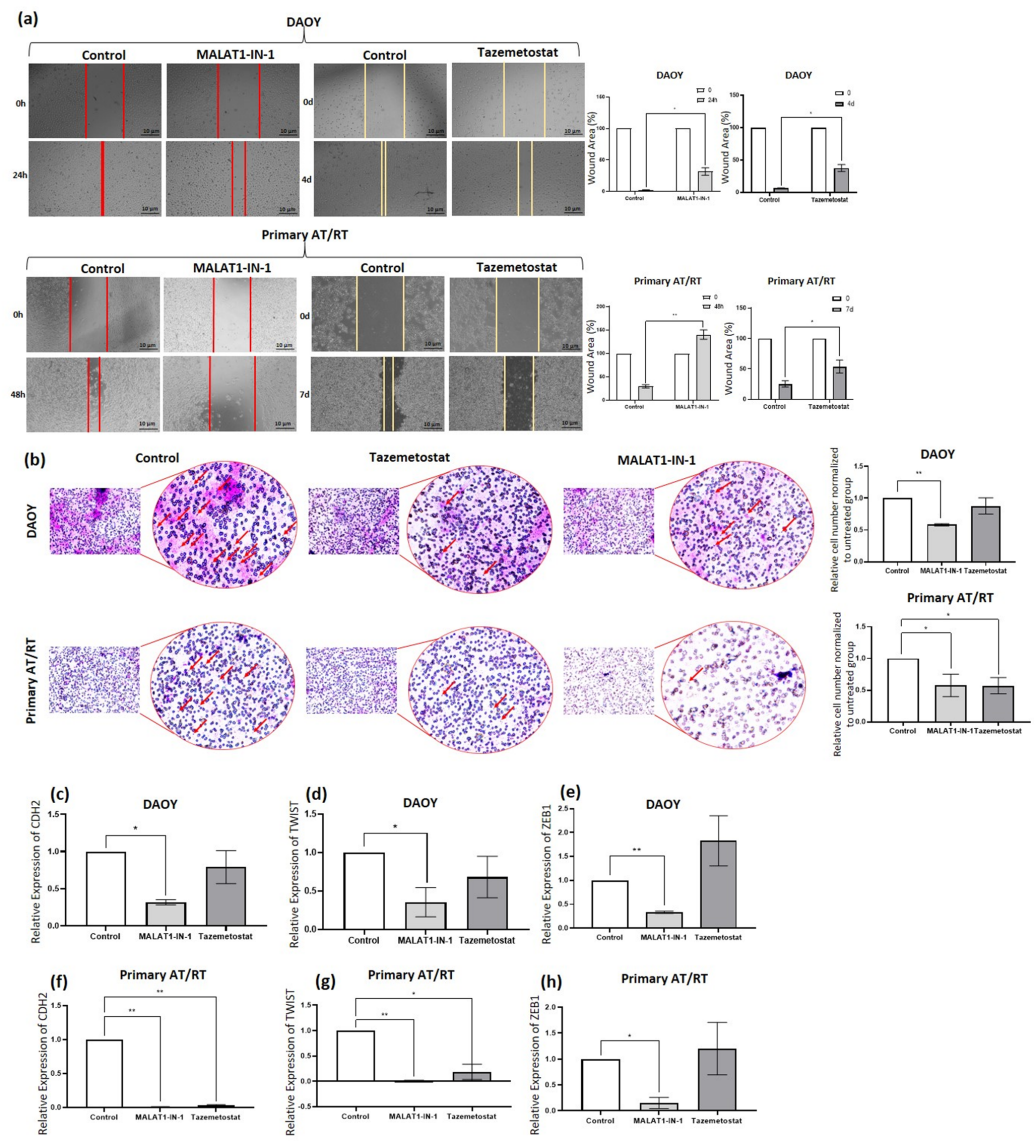
MALAT1 and EZH2 inhibition suppress migration, invasion, and mesenchymal marker expression. DAOY and primary AT/RT cells were treated with tazemetostat (2.5 µM) or MALAT1-IN-1 (5 µM). (**a**) Wound-healing assays showing reduced migration after tazemetostat (4–7 days) or MALAT1-IN-1 (24–48 h) treatment in DAOY and primary AT/RT cells. Wound borders are indicated (red: tazemetostat; yellow: MALAT1-IN-1). Scale bar: 10 μm. *p < 0.05, **p < 0.005. (**b**) Representative Transwell invasion images of DAOY and primary AT/RT cells following treatment with Tazemetostat (2.5 µM) or MALAT1-IN-1 (5 µM). Quantification was performed by counting stained cells in five random fields per replicate. Data represent the mean of three independent experiments normalized to the control ± SD. *p* < 0.05 vs. control. Right panels show higher-magnification insets. Scale bar: 75 μm. *p < 0.05, **p < 0.0001. (**c**, **f**) RT–qPCR analysis of CDH2 expression in DAOY and primary AT/RT cells. (**d**, **e**, **g**, **h**) RT–qPCR analysis of TWIST and ZEB1 expression. All values were normalized to GAPDH. Controls: untreated or vehicle-treated (DMSO) cells. Control values were normalized to 1 by ΔΔCt; error bars omitted. *p < 0.05, **p < 0.0001

The effects of EZH2 and MALAT1 inhibition on tumor aggressiveness were evaluated using migration, invasion, and EMT marker expression assays. In wound-healing assays, both EZH2 and MALAT1 inhibition significantly suppressed DAOY cell migration (*p* < 0.05), whereas MALAT1 inhibition more effectively reduced migration in primary AT/RT cells than EZH2 inhibition (*p* < 0.005 vs. *p* < 0.05; Fig. 4a). In the wound-healing assays, both inhibitors significantly delayed wound closure at 48 and 72 h (Fig. 4). However, it should be noted that at these later time points, the observed results likely represent a combined effect of inhibited cell migration and reduced cell viability, as the inhibitors also significantly impacted the overall proliferation rate of both DAOY and primary AT/RT cells. In transwell assays, MALAT1 inhibition significantly reduced the invasion of DAOY cells (*p* < 0.0001), whereas EZH2 inhibition had no significant effect. In primary AT/RT cells, both inhibitors reduced invasion (*p* < 0.05; Fig. 4b). Consistent with functional assays, EZH2 inhibition did not significantly alter EMT marker expression in DAOY cells, whereas MALAT1 inhibition suppressed CDH2, TWIST, and ZEB1 (*p* < 0.05–*p* < 0.0001; Fig. 4c–e). In primary AT/RT cells, both inhibitors reduced CDH2 and TWIST expression (*p* < 0.05), but only MALAT1 inhibition decreased ZEB1 (*p* < 0.05), aligning with its stronger antimigratory effect (Fig. 4f–h). To validate our findings in an authentic disease context, we utilized patient-derived primary cells. The AT/RT identity of these cells was confirmed by the diagnostic loss of INI1 protein (Fig. 1e). Consistent with the DAOY model, the primary AT/RT cells showed significant MALAT1-EZH2 dependency across proliferation, gene expression, and invasion assays (Figs. 2, 3 and 4).

### MALAT1 and EZH2 inhibition reduce tumorsphere growth and viability

**Fig. 5:**
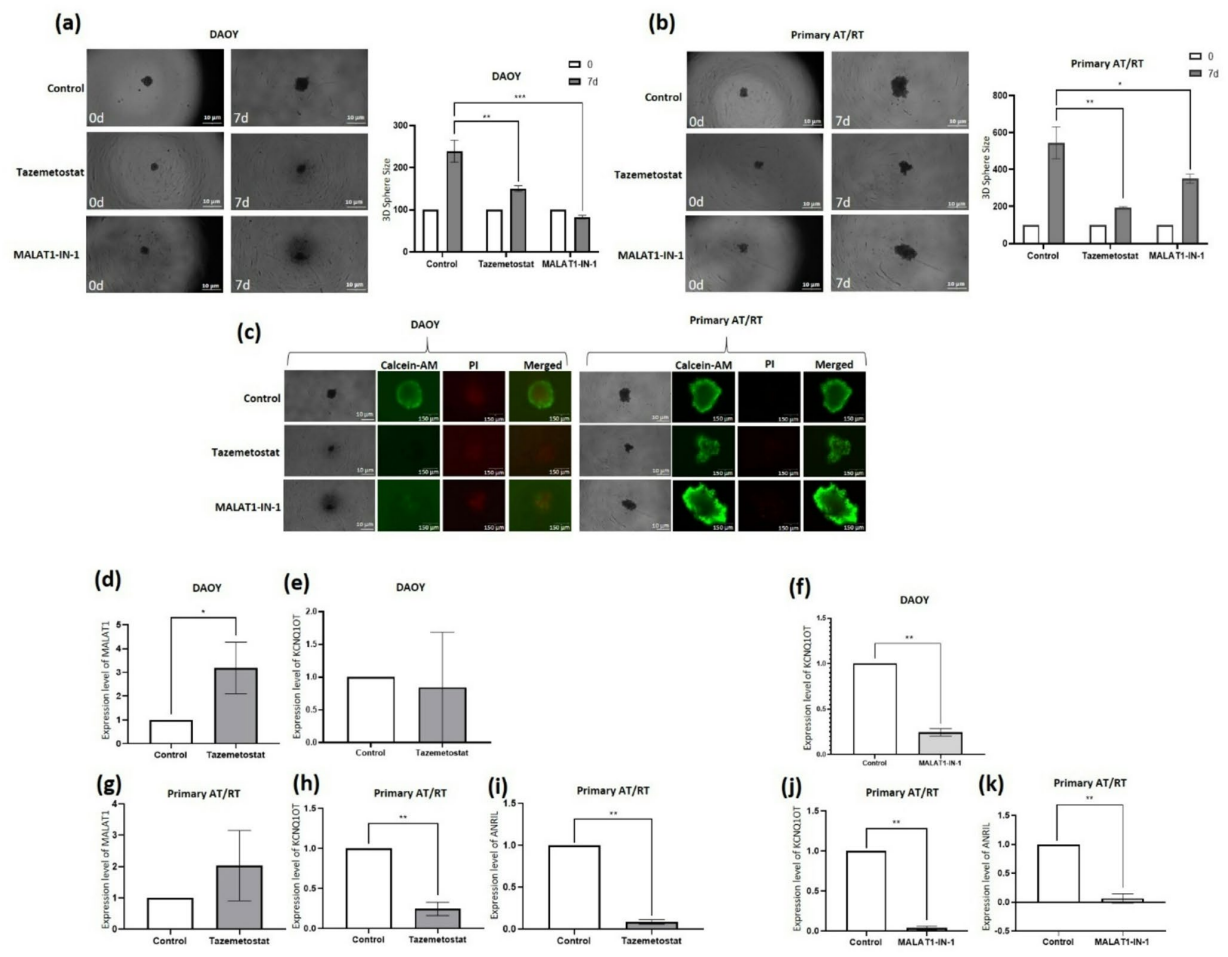
MALAT1 and EZH2 inhibition suppress tumorsphere growth and viability. (**a**,** b**) Representative images of tumorspheres formed by DAOY and primary AT/RT cells at baseline (0 d) and after 7 days of tazemetostat or MALAT1-IN-1 treatment. Scale bar: 10 μm. Sphere size was quantified using ImageJ. (**c**) Live/dead staining of tumorspheres after 7 days of treatment. Calcein-AM–positive cells (green) indicate viable cells, while PI-positive cells (red) indicate necrotic cells. Merged images were acquired at 40× magnification. Scale bar: 150 μm. *p < 0.05, **p < 0.005, ***p < 0.0005. (**d–f**) RT–qPCR analysis of MALAT1 and KCNQ1OT1 expression in DAOY cells following tazemetostat or MALAT1-IN-1 treatment. (**g–k**) RT–qPCR analysis of MALAT1, KCNQ1OT1, and ANRIL expression in primary AT/RT cells following tazemetostat or MALAT1-IN-1 treatment. All values were normalized to GAPDH. Controls: untreated or vehicle-treated (DMSO) cells. Control values were normalized to 1 by ΔΔCt; error bars omitted. *p < 0.05, **p < 0.0001

Tazemetostat significantly suppressed tumorsphere growth in DAOY and primary AT/RT cells over 7 days (*p* < 0.005). MALAT1-IN-1 further reduced sphere growth in primary AT/RT cells (*p* < 0.05) and decreased DAOY sphere size compared with baseline (*p* < 0.0005) (Fig. 5a, b). Both EZH2 and MALAT1 inhibition similarly reduced viability in DAOY spheres, whereas MALAT1 inhibition did not further decrease viability in primary AT/RT spheres compared with EZH2 inhibition (Fig. 5c). Overall, despite assay-specific differences, MALAT1 and EZH2 inhibition consistently suppressed migration, invasion, and tumorsphere growth, supporting their comparable roles in driving AT/RT aggressiveness.

### Effects of PRC2 and MALAT1 inhibition on cancer-related lncRNA expression

The impact of EZH2 and MALAT1 inhibition on MALAT1, ANRIL, and KCNQ1OT1 expression was evaluated. EZH2 inhibition unexpectedly increased MALAT1 expression in DAOY cells (*p* < 0.05; Fig. 5d), while a non-significant increase was observed in primary AT/RT cells (Fig. 5g), suggesting context-dependent regulation of MALAT1 by PRC2. In DAOY cells, MALAT1 inhibition significantly reduced KCNQ1OT1 expression (*p* < 0.0001), whereas EZH2 inhibition had no significant effect (Fig. 5e, f). In primary AT/RT cells, both EZH2 and MALAT1 inhibition significantly decreased KCNQ1OT1 levels (*p* < 0.0001; Fig. 5h, j). ANRIL was undetectable in DAOY cells but was significantly reduced in primary AT/RT cells following both EZH2 and MALAT1 inhibition (*p* < 0.0001; Fig. 5i, k). Overall, PRC2 and MALAT1 inhibition modulated oncogenic lncRNA expression in a cell-type–dependent manner. Notably, MALAT1 upregulation following EZH2 inhibition in DAOY cells suggests that PRC2 targeting may trigger compensatory lncRNA responses, depending on the cellular context.

## Discussion

AT/RT is a highly aggressive pediatric brain tumor where the clinical efficacy of EZH2 inhibitors, such as tazemetostat, is limited by side effects and incomplete responses [9, 26, 27]. This necessitates identifying alternative epigenetic targets. Although non-coding RNAs are key regulators in other brain cancers [28, 29], their role in AT/RT is poorly understood [30, 31]. Pathogenically, SMARCB1 loss disrupts the physiological antagonism between the SWI/SNF and PRC2/EZH2 complexes. This imbalance leads to unrestrained EZH2 activity and subsequent H3K27me3 accumulation at tumor suppressor promoters, causing their transcriptional silencing [4, 22, 27]. Specifically, the loss of SWI/SNF-mediated chromatin remodeling allows PRC2 to dominate the epigenetic landscape, a hallmark of rhabdoid tumor pathogenesis that underscores the therapeutic potential of EZH2 inhibition [4, 17, 22]. Our study identifies MALAT1 as a key epigenetic partner of EZH2 in AT/RT, providing a novel translational target to potentially overcome the limitations of current EZH2-directed therapies.

In this study, we identified high expression of MALAT1, ANRIL, and KCNQ1OT1 in AT/RT patient tissues. Elevated levels of these lncRNAs were correlated with unfavorable clinicopathological features, such as intermediate tumor localization and a high Ki67 proliferation index (≥ 50%). Importantly, our findings revealed that MALAT1 interacted with EZH2, a catalytic subunit of the PRC2 complex, in primary AT/RT cells. This interaction promotes H3K27 trimethylation (H3K27me3), a hallmark of PRC2-mediated transcriptional repression [11, 21, 22]. Inhibition of MALAT1 significantly reduces H3K27me3 levels, particularly in primary AT/RT cells, where aberrant H3K27me3 formation is strongly linked to prognosis. Our immunoblotting data confirmed that MALAT1 suppression not only impairs H3K27me3 but also leads to a reduction in EZH2 protein levels, suggesting a potential protein-stability regulatory role. These results suggest that MALAT1 not only modulates PRC2 activity, but may also help sustain EZH2 expression, thereby reinforcing its oncogenic function in AT/RT. These results highlight MALAT1 as a novel regulator of PRC2 activity in AT/RTs.

Functionally, MALAT1 inhibition suppressed the proliferation, migration, and invasion of primary AT/RT cells more effectively than EZH2 inhibition did. While targeting EZH2 or MALAT1 produced comparable antimigratory effects in DAOY cells, MALAT1 inhibition showed superior antimigratory and anti-invasive activities in primary AT/RT cells. It is also possible that the apparent enlargement of the wound area observed in AT/RT cells treated with MALAT1-IN-1 at later time points may be partially due to reduced cell viability induced by MALAT1 inhibition in addition to its antimigratory effect. Furthermore, three-dimensional tumor sphere models confirmed that MALAT1 suppression reduced tumor growth and stem cell properties in DAOY and AT/RT cells. These findings collectively suggest that MALAT1 is not only a mediator of PRC2 activity but also contributes to PRC2-independent oncogenic pathways, in line with previous evidence that MALAT1 regulates cellular functions such as nuclear–cytoplasmic protein transport and TGF-β-associated signaling [32]. In this study, although the magnitude of the inhibitory effects varied between cell types and functional assays, inhibition of both MALAT1 and EZH2 consistently impaired the aggressive tumor phenotypes. Comparative analyses revealed that EZH2 inhibition using tazemetostat led to a compensatory increase in MALAT1 expression, particularly in DAOY cells, potentially limiting its therapeutic efficacy. In contrast, MALAT1 inhibition suppresses the expression of ANRIL and KCNQ1OT1, two other lncRNAs associated with malignant prognosis [12–14, 33], indicating that MALAT1 inhibition may confer broader therapeutic benefits by modulating multiple oncogenic lncRNAs. Furthermore, the compensatory upregulation of MALAT1 observed following tazemetostat treatment in DAOY cells reveals a potential feedback loop that might contribute to EZH2-inhibitor resistance. This provides a strong therapeutic rationale for dual targeting of the MALAT1–EZH2 axis. This feedback mechanism underscores the potential for MALAT1-targeted agents to sensitize AT/RT cells to EZH2 inhibition. The observed upregulation of MALAT1 in response to EZH2 inhibition suggests a feedback or compensatory mechanism that may allow cells to maintain certain PRC2-related functions. This finding underscores the potential limitation of single-agent epigenetic therapies. Future studies focusing on the dual inhibition of the MALAT1-EZH2 axis are warranted to determine if blocking both components can achieve a synergistic therapeutic effect and prevent the compensatory activation of lncRNAs, thereby offering a more robust treatment strategy for pediatric AT/RT.

While our retrospective cohort of ten patients and use of patient-derived primary cells provide biologically relevant insights, we acknowledge the limitations inherent to studying such a rare pediatric malignancy. The reliance on RT-qPCR and FFPE tissues reflects the constraints of clinical samples; however, the clear *SMARCB1* loss and H3K27me3 reduction serve as robust surrogates for the biological activity we describe. Crucially, as our functional claims are based on in vitro systems, future studies utilizing orthotopic xenograft models will be essential to account for systemic complexities and validate the in vivo efficacy of MALAT1 targeting. While our study focused on the targeted regulation of key cell cycle and mesenchymal markers by the MALAT1-EZH2 axis, global transcriptomic approaches such as RNA-seq could be employed in future studies to fully map the extensive gene networks influenced by this interaction in the context of SMARCB1 deficiency. Despite the novel insights provided by this study, some limitations should be acknowledged. First, the use of the DAOY cell line a medulloblastoma-derived model was intended to explore shared epigenetic vulnerabilities; however, it may not perfectly represent the AT/RT-specific molecular environment. Second, while our use of patient-derived primary AT/RT cells enhances clinical relevance, these cells were obtained from a single patient. Given the known inter-patient heterogeneity in AT/RT, our findings regarding the MALAT1-EZH2 axis should be interpreted as a mechanistic proof of concept. Future studies utilizing larger multi-center cohorts and orthotopic xenograft models will be essential to validate the in vivo efficacy of MALAT1 targeting and its synergy with existing epigenetic drugs.

This study identifies MALAT1 as highly expressed in AT/RT and directly interacts with the EZH2/PRC2 complex. In patient-derived cells, MALAT1 inhibition reduced H3K27me3 levels, suppressed oncogenic phenotypes (proliferation, migration, invasion, tumor sphere growth), and limited stemness. MALAT1 suppression showed stronger or complementary antitumor effects compared to EZH2 inhibition and downregulated other poor-prognosis lncRNAs (ANRIL, KCNQ1OT1). These results highlight MALAT1 as a promising therapeutic target for AT/RT, potentially in combination with EZH2 inhibition. Future studies should include larger patient cohorts, validation in animal models, and multi-omics profiling to fully characterize MALAT1’s oncogenic role and evaluate its potential in combination therapies. Collectively, our findings provide a compelling rationale for integrating MALAT1-targeted strategies into the burgeoning landscape of epigenetic therapies for pediatric rhabdoid tumors.

## Supplementary Information

Below is the link to the electronic supplementary material.

Supplementary Material 1

## Data Availability

All the data and materials generated and/or analyzed during the current study are available from the corresponding author upon reasonable request.
