# Supplementary material for: The MALAT1-EZH2 axis regulates PRC2 activity and promotes the mesenchymal phenotype in pediatric atypical teratoid/rhabdoid tumors
Source: J Neurooncol. 2026 Mar 31;177(2):83. doi: 10.1007/s11060-026-05539-x (PMC13038470; doi:10.1007/s11060-026-05539-x)
Supplement: Supplementary file 1 — Supplementary Material 1 [file 11060_2026_5539_MOESM1_ESM.docx]

**Supplementary Table 1.** Summary of demographic data, tumor localization, and expression levels of Ki-67 and MALAT1 in the primary AT/RT cases.

| **Patient ID** | **Age / Gender** | **Tumor Location** | **Ki-67 Index (%)** | **MALAT1 Expression (Relative)** | **INI-1 (SMARCB1) Status** |
| --- | --- | --- | --- | --- | --- |
| P1 | 2 Years / M | - | 400/1000 | 7.32 | Loss of Expression |
| P2 | 20 Years / M | Lateral Ventricle | 380/1000 | 2.66 | Loss of Expression |
| P3 | 18 Months / F | 3. Ventricle | 400/1000 | 1.36 | Loss of Expression |
| P4 | 4 Months / F | 4. Ventricle | 300/1000 | 4.99 | Loss of Expression |
| P5 | 3 Months / F | 4. Ventricle | 1/1000 | 4.36 | Loss of Expression |
| P6 | 4 Months / M | 3. Ventricle | 500/1000 | 27.87 | Loss of Expression |
| P7 | 3 Months / M | 3. Ventricle | 800/1000 | 1.6 | Loss of Expression |
| P8 | 17 Months / M | 4. Ventricle | 750/1000 | 17.2 | Loss of Expression |
| P9 | 9 Months / F | Right Temporal+4.Ventricle | 20/1000 | 4.38 | Loss of Expression |
| P10 | 19 Months / M | Right Frontal | Unknown | 1.7 | Unknown |

Abbreviations: P, Patient; M, Male; F, Female; Ki-67, Proliferation index; MALAT1, Metastasis-associated lung adenocarcinoma transcript 1; INI-1, Integrase interactor 1 (SMARCB1).

Note: Expression levels for MALAT1 were determined via RT-qPCR. "Unknown" indicates clinical or pathological data that were unavailable in the retrospective archival records.
